# Effect of Temperature and Humidity on Oil Quality of Harvested *Torreya grandis* cv. Merrillii Nuts During the After-Ripening Stage

**DOI:** 10.3389/fpls.2020.573681

**Published:** 2020-10-23

**Authors:** Zuying Zhang, Hangbiao Jin, Jinwei Suo, Weiyu Yu, Minyin Zhou, Wensheng Dai, Lili Song, Yuanyuan Hu, Jiasheng Wu

**Affiliations:** ^1^State Key Laboratory of Subtropical Silviculture, Zhejiang A&F University, Lin’an, China; ^2^Zhuji Academy of Forestry, Zhuji, China; ^3^Sino-Australia Plant Cell Wall Research Centre, School of Forestry and Biotechnology, Zhejiang A&F University, Lin’an, China

**Keywords:** *Torreya grandis*, post-harvest ripening stage, temperature, relative humidity, unsaturated fatty acids, oil quality

## Abstract

Temperature and relative humidity (RH) influence post-harvest ripening, a crucial stage for quality promotion in some oil plants or fruits. *Torreya grandis* cv. Merrillii nuts, which are rich in unsaturated fatty acids (UFA), are easily affected by temperature and humidity, and they oxidize quickly during the post-harvest ripening stage, leading to the deterioration of nut quality. In this study, the main nutraceutical components, fatty acid composition, and related metabolic parameters of lipid rancidity under four treatments (20°C and 70% RH, T20-LH; 30°C and 70% RH, T30-LH; 20°C and 90% RH, T20-HH; 30°C and 90% RH, T30-HH) were measured. The post-harvest ripening process was advanced under HH treatments (T20-HH and T30-HH) compared to LH treatments (T20-LH and T30-LH) and was associated with a shorter time for the seed coat to turn dark black and a faster reduction in starch content. The amount of unsaturated fatty acids significantly increased under the T20-HH treatment, but significantly decreased under the T30-HH treatment from 12 to 16 d of ripening time. The acid value (AV) and lipase activity under the T30-HH treatment remained virtually constant from 12 to 16 d of ripening time, and this was accompanied by a dramatic increase in peroxide value (POV), lipoxygenase (LOX) activity, and relative expression of the *LOX2* gene. Meanwhile, a significant positive correlation between LOX activity and POV, malondialdehyde (MDA) content, and O_2_⋅^–^ content was observed. The results imply that the lower amount of oxidative rancidity induced by the T20-HH treatment is related to the LOX activity induced by down-regulation of the *LOX2* gene during the late after-ripening stage. Therefore, the T20-HH treatment not only promoted the post-harvest process of *T. grandis* ‘Merrillii’ nuts but also delayed lipid oxidation, which was ultimately associated with better oil quality at the late after-ripening stage.

## Introduction

*Torreya grandis* cv. Merrillii, an evergreen coniferous tree with significant economic value in China, has been listed as a national key protected wild plant species (Shen et al., 2014). *T. grandis* nuts are popular in China because of their good flavor, nutrient-rich properties and high oil content (approximately 42–54%). In addition, unsaturated fatty acids (UFAs) account for the vast majority (approximately 80%) of the total oil content and are mainly composed of oleic acid (C_18:1_), linoleic acid (C_18:2_), and sciadonic acid (C_20:3_), making *T. grandis* unique among other nuts (Ni and Shi, 2014; Wu et al., 2018). The demand for *T. grandis* seeds has been increasing; consequently, this species is now being cultivated in various locations in China (Chen et al., 2008). The cultivation area of *T. grandis* has rapidly expanded, increasing approximately 11.5-fold from 2000 to 2012 (He et al., 2013). *T. grandis* nuts are different from other tree nuts, such as hickory and walnut, in that they need a post-harvest ripening stage for oil accumulation and favorable fatty acid composition before roasting (Ye et al., 2017).

Previous studies have suggested that the ripening process of oil nuts involves nutrient conversion, oil accumulation, and an increase in UFA content (Conde et al., 2008). However, UFA-enriched nuts can easily become rancid, which is associated with undesirable changes in flavor, color, odor, and nutritional value and which could be harmful to human health (Ahmed and Young, 1982; Braddock et al., 1995; Turner et al., 2006; Buranasompob et al., 2007). Environmental conditions, such as light, temperature, relative humidity (RH), and oxygen concentration, can influence the development of rancidity generated by lipid oxidation. Temperature and RH are mainly responsible for the lipid peroxidation, leading to quality deterioration of nuts (Mexis et al., 2009; Onilude et al., 2010). Post-harvest ripening of *T. grandis* nuts is a process that depends on time and the environment, specifically temperature and RH, and involves the conversion of nutrients and the accumulation of oil and UFA in the nuts (Ye et al., 2017). The UFA content of *T. grandis* nuts increases significantly during the post-harvest ripening stage and may be susceptible to lipid oxidation, especially under improper post-ripening conditions. Consequently, it is necessary to investigate the trend of UFA content of *T. grandis* kernels during the post-harvest ripening stage and how it is affected by the conditions of the ripening environment.

Reports have suggested that the oxidation and hydrolyzation of UFA level in oilseeds is closely related to phospholipid-degrading enzymes. Lipase and lipoxygenase (LOX) are enzymes that are mainly responsible for the rancidity of UFA in nut kernels (Rogiers et al., 1998; Chen, 2008). It has been reported that high lipase activity caused rapid oil acidification and release of free fatty acids in the post-harvest oil palm fruit mesocarp (Morcillo et al., 2012). Greater reduction of LOX activity of walnut and almond kernels was observed in short-time heat treatments, delaying the development of oxidative rancidity and extending their shelf-life (Buranasompob et al., 2007). Under the action of LOX, lipid degradation produces many peroxidation products, such as malondialdehyde (MDA) and oxy-radicals (e.g., hydrogen peroxide (H_2_O_2_) and superoxide anion (O_2_−) (Lynch and Thompson, 1984; Rogiers et al., 1998). The production of these compounds directly leads to deterioration of oil quality (Zhang et al., 2012). Soybean seeds produced high levels of MDA during storage at high temperature and high RH (Robert et al., 1980). Fortunately, nut tissue could defend itself against oxidative stress by inducing the gene activity or expression of active oxygen scavengers such as antioxidant enzymes, including superoxide dismutase (SOD, EC 1.15.1.1), peroxidase (POD, EC 1.11.1.7), and catalase (CAT, EC 1.11.1.6) (Ren, 2001; Apel and Hirt, 2004).

Many studies have been performed on the responses of oil quality to different temperature treatments or different humidity treatments in UFA-rich nuts (Onilude et al., 2010; Wen et al., 2017; Ye et al., 2017; Zhou et al., 2019). However, few studies have investigated the interactive influence of temperature and RH on post-harvest process and oil quality. Therefore, efforts must be dedicated to the study of the conversion of nutrients and the quality of *T. grandis* cv. ‘Merrillii’ (later also *Torreya*) nut kernels under different conditions to determine the optimum post-harvest conditions for obtaining high quality nuts. Accordingly, we set out to analyze whether post-harvest storage conditions at different temperatures and RH levels directly advance the post-harvest ripening process in terms of the color and texture of the inter-seed coat and the main nutraceutical components (including starch, soluble sugar, protein, and oil) of *T. grandis* nut kernels. A second goal of this study was to determine the impact of temperature and RH on the associated oil oxidative rancidity metabolism, including lipid oxidative enzymes, peroxidation products, and lipid antioxidative enzymes, and the relative expression of rancidity-associated enzyme genes. Moreover, the relationship between nutraceutical composition and oil rancidity metabolism of nuts was analyzed by the multivariable Pearson correlation. The findings of this study will help to clarify the optimal controlled storage conditions, which are important for the formation of good quality *T. grandis* nut kernels during the post-harvest ripening process, as well as provide a theoretical foundation for later production and processing.

## Materials and Methods

### Plant Materials and Treatments

The *T. grandis* cv. ‘Merrillii’ nuts used in this study were obtained from commercial orchards in Xinchang City, Zhejiang Province, China (29°29′N, 120°54′E) in September 2017. *T. grandis* seeds from outer crown of at least 10 trees at a height of around 1.5–2.0 m were labeled when the seeds emerged from the seed scale (15 April 2017). Seeds of approximately the same size were selected for experiment materials. When the nut aril cracked (mature stage, about 525 d after bloom), the nuts were picked by hand in the morning and transported to the laboratory within 4 h. Only healthy nuts, i.e., those with no infection or physical damage, were selected. The experimental design was a split-plot in randomized complete blocks (three replications) with two values of RH as the main plot and two temperatures as the subplot. Ten kilograms (about 7600 seeds) of *T. grandis* nuts was placed in a thermostat (Eshengtaihe Ctrl Tech B6-12, China), which was a 35 cm × 35 cm × 15 cm cube, as a replicate. There were two temperature conditions (20°C ± 2°C and 30°C ± 2°C) and two RH levels (70% RH ± 2% and 90% RH ± 2%) for a total of four treatments: T20-LH (20°C ± 2°C and 70% RH), T30-LH (30°C ± 2°C and 70% RH), T20-HH (20°C ± 2°C and 90% RH) and T30-HH (30°C ± 2°C and 90% RH). The interior seed coat outside the kernel had completely changed from reddish-brown to black during the after-ripening stage (Shan et al., 2019). In this study, the after-ripening stage completed on the 16th day. Nut samples were taken from the chamber at 4-d intervals during storage for measurements of nutritional components (starch, sugar, protein, and oil); fatty acid; lipid peroxidation; O_2_^–^ production rate; H_2_O_2_ content; SOD, CAT, and POD activity. The shelled nut kernels stored at −80 °C. Data were analyzed from three biological replicates (representing thirty nuts) for each time point.

### Observation of the Interior Coat Color of the Kernel, Moisture Content, and Its Microstructure

Observation of the interior seed coat color of kernels: 4–5 *T. grandis* nuts from each of the four treatments were shelled for color observation of the kernel coat at 4-d intervals during post-harvest ripening storage. The moisture content was measured by oven drying 10 g of kernels at 103°C for 3 h, followed by a further period of 48 h at 60°C until a constant weight was achieved.

(1) Moisturecontent(%)=100×(freshweight-dryweight)/fresh⁢weight

Observation of kernel transection microstructure: the distance between the kernel and the interior coat was determined by the method reported by Ye et al. (2017) with some modifications. The *T. grandis* kernels with an interior seed coat were fixed with a formalin-acetic acid-alcohol mixture for at least 24 h, then they were sliced according to the paraffin section method. Transverse sections, 8 μm thick, were cut with a rotary microtome and double-stained with Safranine-Fast Green (Yuanye Biotechnology Co. Shanghai, China). The distance between the kernel and the interior seed coat was determined through photomicrographs taken with a light microscope (Olympus DP 70, Tokyo, Japan).

### Measurement of Oil Content, Soluble Sugar, Starch, and Soluble Protein

Oil content was determined by the methods reported by García-Ayuso et al. (2000) with minor modifications. The oil was extracted in a Soxhlet apparatus with petroleum ether solvent (boiling point range, 30–60°C) for 8–12 h. After being defatted, the samples were dried overnight (10–12 h) in a fume hood to remove residual petroleum ether and weighed to calculate lipid content.

(2) Oilcontent=(dryweightbeforedefatting-dryweightafterdefatting)/dryweightbeforedefatting.

Next, the samples of nut residue were used for the measurement of soluble sugar, starch, and soluble protein. Both the soluble sugar content and the amount of starch were quantified according to the method of Zheng (2006), with slight modifications. Ten mL of distilled water was added to the residue (without soluble sugar) and boiled for 15 min. After cooling, 20 mL of perchloric acid was added, and the sample was left to stand for 15 min at room temperature. The extraction was centrifuged at 10,000 *g* for 15 min, and the supernatant was transferred to a 100 mL volumetric flask. This extraction procedure was performed in triplicate; all the supernatants were combined into the 100 volumetric flask to make a 100 mL solution for assays. Then, 1 mL of the sample solution and 5 mL of anthrone reagent were added, in turn, into the tubes, which were placed in a boiling water bath for 10 min. After cooling, they were placed in the dark for 10 min to test the absorbance at 620 nm. Standard glucose solutions were used to develop a calibration curve. Soluble protein content was measured according to the Coomassie Brilliant Blue G-250 method (Read and Northcote, 1981). The soluble protein content was measured by a spectrophotometer at 595 nm. The oil, soluble sugar, starch, and soluble protein content were expressed as g kg^–1^ dry weight.

### Measurement of Fatty Acid Composition

Fatty acid methyl esters (FAME) were prepared according to the method used by Park and Goins (1994), with some modifications. An aliquot of 60 mg of *T. grandis* kernel oil was dissolved in 4 mL iso-octane and methyl esterified with 200 μL of 2 M (w/v) KOH (formulated with methanol). The sample was thoroughly shaken for 30 s, and then 1 g of NaHSO_4_ was added to neutralize KOH. The supernatant was collected and subjected to gas chromatographic analysis.

The analysis was performed on a gas chromatograph (Thermo Scientific TRACE 1300, Thermo Fischer Scientific, Waltham, MA, United States) equipped with a 30 m × 0.25 mm × 0.25 μm capillary column (HP-INNOWAX, Agilent Technologies, Santa Clara, CA, United States). The injector port temperature was maintained at 220°C. Split injections were performed with a 20:1 split ratio. The injection volume was set at 1 mL. Column heating was performed; it started at 140°C, increased to 250°C at a rate of 4°C per min, and was held at 250°C for 2 min. The flow rate of hydrogen and air was 30 and 400 mL min^–1^, respectively.

Fatty acid methyl esters was identified by comparison of retention time of the gas chromatographic peaks with those of commercial-free fatty acid methyl ester standards (Sigma-Aldrich, St. Louis, MO, United States). They were automatically computed as a percentage by the data processor from the ratio of individual peak area to the total peak area of fatty acids. The results were expressed as g kg^–1^ dry weight.

Then, the ratio of unsaturated fatty acids (UFAs) to saturated fatty acids (SFAs) was calculated using the following formula:

(3) $$U\,F\,A/S\,F\,A=\frac{\left[C_{18:1}\right]+\left[C_{18:2}\right]+\left[C_{20:2}\right]+\left[C_{20:3}\right]}{\left[C_{16:0}\right]+\left[C_{18:0}\right]}$$

where C_18:1_ is the content of oleic acid (g kg^–1^); C_18:2_ is the content of linoleic acid (g kg^–1^); C_20:2_ is the content of eicosadienoic acid (g kg^–1^); C_20:3_ is the content of sciadonic acid (g kg^–1^); C_16:0_ is the content of palmitic acid (g kg^–1^); and C_18:0_ is the content of stearic acid (g kg^–1^).

### Measurement of Rancidity-Associated Enzymes and Oxidation of Oil Lipase and LOX Activity

The activity of lipase was determined by a modified method of that used by Asperen (1962). A mass of 4 g of the fresh kernel was ground to a fine powder and extracted with 25 mL of 25 mM phosphate buffer containing 0.05 M mercaptoethanol and centrifuged for 10 min at 10,000 *g* at a temperature of 4°C. The supernatant was collected and then used as the crude enzyme extraction. The reaction mixture consisted of 2.3 mL of 0.2 M phosphate buffer (pH 7.8), 0.5 mL of 0.2 mM a-naphthyl acetate dissolved in 10% acetone, and 0.2 mL enzyme solution. After incubation for 30 min at 25°C, 1 mL of 0.15% Fast Blue RR was added. The control contained 2.5 mL of 0.2 M phosphate buffer (pH 7.8) and 0.5 mL of 0.2 mM a-naphthyl acetate solution.

LOX activity was assayed according to the method previously described by Mizuno et al. (2003). A mass of 4 g of flesh tissue was ground to a powder in liquid nitrogen, homogenized with 25 mL of 50 mM Tris–HCl (pH 8.0), and centrifuged for 10 min at 10,000 *g* at 4°C for LOX extraction. The reaction mixture (3 mL) contained 2.8 mL of 50 mM sodium phosphate buffer (pH 7.0), 0.1 mL of 10 mM sodium linoleic acid solution, and 0.1 mL of extract. The reaction mixture without the enzyme solution was used as the control.

A total of 1 U of lipase and LOX activity was defined as an absorbance change of 0.1 units per min. The specific lipase and LOX activity were expressed on a protein basis as U mg^–1^.

### Acid Value and Peroxide Value

The acid value (AV) and peroxide value (POV) were determined according to standard methods (Fu et al., 2016). The AV and POV were expressed as g kg^–1^ oil and meq kg^–1^ oil, respectively.

### Expression Analysis of *Lipase* and *LOX2* Genes

Real-time quantitative PCR analysis was conducted on *lipase* and *LOX* genes, which were selected based on RNA-Seq data (Wu et al., 2018). Twenty nuts from each treatment were collected for RNA extraction using the 74904 Rneasy Plant Mini Kit (Tiangen, Beijing, China). First-strand cDNA synthesis was carried out with 3 μg of purified total RNA using PrimeScript RT Reagent Kit Perfect Real Time (Takara, Dalian, China). Forward and reverse specific primers were designed for four deferentially expressed genes (Unigene1066989 and Unigene1025506). Fusion-specific and precursor-specific oligonucleotide primers are listed in Supporting Information. The ACTB gene was used as a control in the described experiments. The primers of *lipase*, *LOX2*, and *ACTB* genes are listed in Table S1. PCR amplifications were performed in a 20 μL reaction volume with the ChamQ SYBR qPCR Master Mix (Vazyme, Nanjing, China) using the Roche LightCycler 480 System (Roche, Basel, Switzerland). PCR involved a 95°C step held for 30 s, followed by 45 cycles at 95°C for 10 s, melting temperature (depending on primer Tm value) for 10 s, 72 °C for 20 s, and 72°C for 2 min. The relative expression was calculated by the formula 2^–ΔΔ*Cp*^. The experiment was performed with three biological replications.

### Lipid Peroxidation

The lipid peroxidation level in the *T. grandis* nut kernel tissue was determined in terms of MDA content by the method of Chen et al. (2013). Flesh tissue (2.0 g) was ground in an ice bath with 10 mL of 10% (w/v) trichloroacetic acid (TCA), and then was centrifuged at 10,000 *g* for 10 min at 4°C. Three milliliters of the supernatant was added to 3 mL of 0.5% (w/v) thiobarbituric acid (formulated with 10% TCA), mixed and heated at 95°C for 20 min, immediately cooled to room temperature, and centrifuged at 10,000 *g* for 10 min at 4°C. The absorbance of the supernatant was recorded at 532, 600, and 450 nm with a spectrophotometer (UV-2550, Shimadzu, Kyoto, Japan). The content of MDA was expressed on a dry weight basis as nmol g^–1^.

### Superoxide Anion and Hydrogen Peroxide

The amount of superoxide anion (O_2_^.–^) in the *T. grandis* kernels tissue was determined by the method of hydroxylamine oxidation and carried out according to Chen et al. (2013). Briefly, 0.5 mL extract was incubated with 0.5 mL of 65 mM K_2_PO_4_ buffer and 0.1 mL of 10 mM hydroxylamine hydrochloride at 25 °C for 20 min. Then, the incubated solution was mixed with 1 mL of 58 mM sulfonamide and 1 mL of 7 mM naphthylamine and further kept for 10 min at 25 °C. The absorbance was recorded at 530 nm. The content of O_2_^–^- was expressed on a dry weight basis as nmol g^–1^ min^–1^.

The amount of hydrogen peroxide (H_2_O_2_) was measured by the method reported in Brennan and Frenkel (1977). The extract (1 mL) was mixed with 0.1 mL of 5% Ti(SO_4_)_2_ and 0.2 mL of concentrated NH_4_OH solution. The titanium-peroxide complex precipitated, and this sediment was dissolved in 4 mL of 2 M H_2_SO_4_ after centrifugation at 3,000 *g* for 10 min. The absorbance was measured at 415 nm. The content of H_2_O_2_ was expressed on a dry weight basis as mM g^–1^.

### Measurement of Antioxidant Enzymes

Superoxide dismutase (SOD, EC 1.15.1.1) activity was assayed by measuring its ability to inhibit the photoreduction of nitroblue tetrazolium (NBT) according to Dhindsa et al. (1981). One unit of SOD activity was defined as the amount of enzyme required to produce 50% inhibition of the reduction of NBT at 560 nm. Activities of peroxidase (POD, EC 1.11.1.7) and catalase (CAT, EC 1.11.1.6) were determined using the methods of Fu and Huang (2001). For POD, the oxidation of guaiacol was measured by the increase in absorbance at 470 nm for 120 s. For CAT, the decomposition of H_2_O_2_ was measured by the decline in absorbance at 240 nm for 60 s. A total of 1 U of POD and CAT activity was defined as an absorbance change of 0.1 units per min. The activity of each enzyme was expressed on a protein basis as U mg^–1^.

### Statistical Analysis

The significance of RH, temperature, and after-ripening time was estimated by the analysis of variance (ANOVA). The effects of RH, temperature, and after-ripening time on each variable were evaluated with a three-way ANOVA without initial samples (0 d). Data are presented as the mean ± standard deviation.

## Results

### Effects of Post-harvest Ripening Conditions on Appearance, Moisture Content, and Microstructure of *T. grandis* Nuts

**FIGURE 1:**
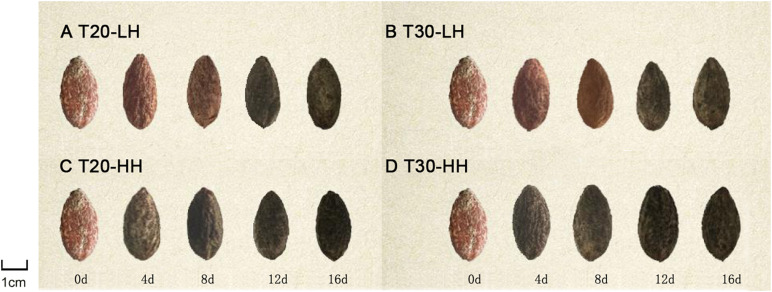
External changes in dehulled *T. grandis* cv. ‘Merrillii’ nuts during post-harvest ripening at different temperature and relative humidity (RH) conditions. Note: **(A)**: T20-LH, at 20°C and 70% RH; **(B)**: T30-LH, at 30°C and 70% RH; **(C)**: T20-HH, at 20°C and 90% RH; **(D)** T30-HH, at 30°C and 90% RH.

The color of the interior seed coat of *T. grandis* kernels on 0 d during ripening time was reddish and gradually turned to dark black during the after-ripening stage (Figure 1). The color of the interior seed coat of *T. grandis* turned dark black at 8 d and 12 d under HH and LH treatments of after-ripening time, respectively.

**FIGURE 2:**
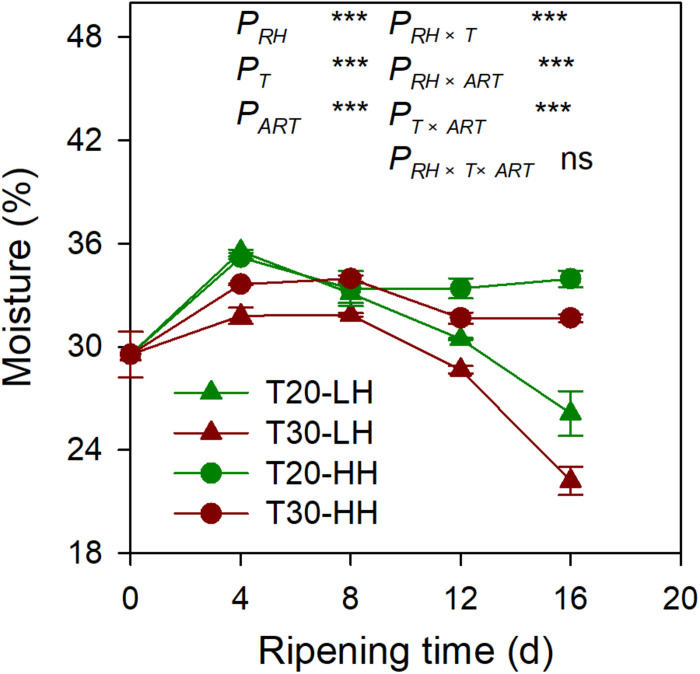
The moisture of *T. grandis* cv. ‘Merrillii’ nuts during post-harvest ripening at different temperature and relative humidity (RH) conditions. *P*_*RH*_, RH effect; *P*_*T*_, temperature effect; *P*_*ART*_, after-ripening time. *P_*RH*_*_×_ *_*T*_*, RH × temperature interaction effect; *P_*RH*_*_×_ *_*ART*_*, RH × after-ripening time interaction effect; *P_*T*_*_×_ *_*ART*_*, temperature × after-ripening time interaction effect; *P_*RH*_*_×_ *_*T*_*_×_ *_*ART*_*, RH × temperature × after-ripening time. Error bars represent standard error based on three biological replicates. Asterisks denote significant differences using Student’s *t*-test, *P < 0.05, **P < 0.01, and ***P < 0.001. ns stands for not significant.

The moisture content of the kernel of four different treatments showed a decreasing trend from 4 to 16 d of after-ripening time (Figure 2). The moisture content of kernels significantly decreased by 26.5% and 30.2% under the T20-LH and T30-LH treatments from 4 to 16 d of after-ripening time, respectively, and the moisture significantly decreased by 3.5% and 5.8% under the T20-HH and T30-HH treatments, respectively.

**FIGURE 3:**
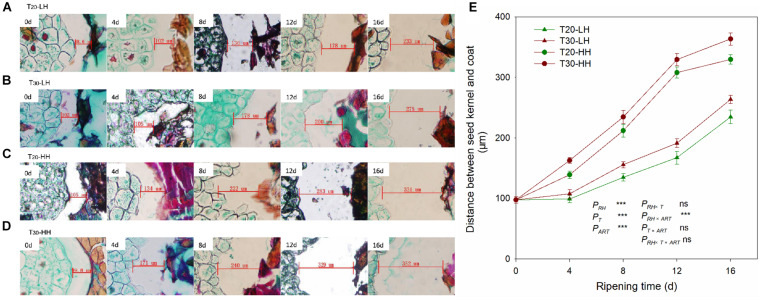
Seed kernel transection microstructure **(A–D)** and the changes in distance between the seed kernel and coat **(E)** of *T. grandis* cv. ‘Merrillii’ nuts during post-harvest ripening at different temperature and relative humidity (RH) conditions. *P*_*RH*_, RH effect; *P*_*T*_, temperature effect; *P*_*ART*_, after-ripening time. *P_*RH*_*_×_ *_*T*_*, RH × temperature interaction effect; *P_*RH*_*_×_ *_*ART*_*, RH × after-ripening time interaction effect; *P_*T*_*_×_ *_*ART*_*, temperature × after-ripening time interaction effect; *P_*RH*_*_×_ *_*T*_*_×_ *_*ART*_*, RH × temperature × after-ripening time. Error bars represent standard error based on three biological replicates. Asterisks denote significant differences using Student’s *t*-test, *P < 0.05, **P < 0.01, and ***P < 0.001. ns stands for not significant.

The distances between the kernel and the interior seed coat under the four treatments increased rapidly with increasing after-ripening time (Figure 3). The cell wall of interior seed coat was intact on 0 d, and then it degraded with increasing after-ripening time (Figures 3A–D). The distance between the kernel and the interior seed coat under the HH treatments (T20-HH and T30-HH) was significantly larger than that of the LH treatments (T20-LH and T30-LH) (Figure 3E).

### Effects of Different Post-harvest Ripening Conditions on Main Nutraceutical Components of *T. grandis* Nuts

**FIGURE 4:**
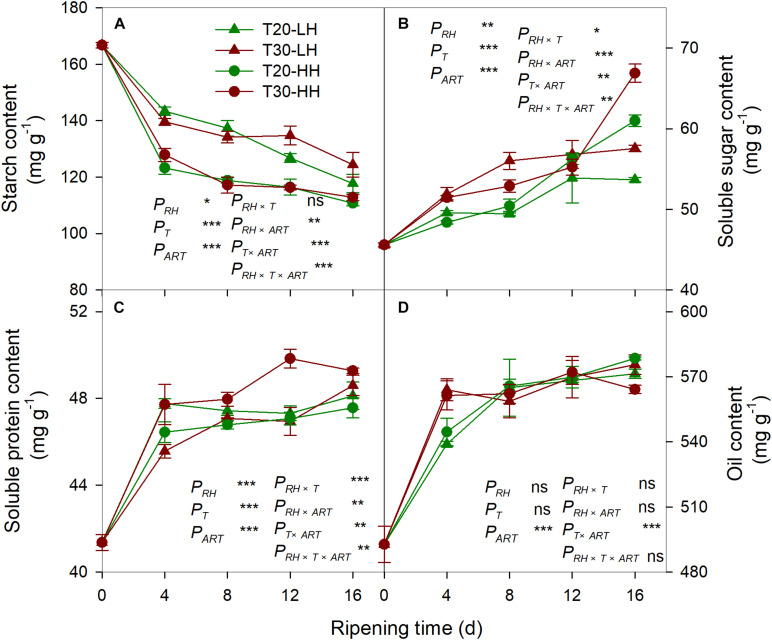
Content of starch **(A)**, soluble sugar **(B)**, soluble protein **(C)**, and oil **(D)** in *T. grandis* cv. ‘Merrillii’ nuts during post-harvest ripening at different temperature and relative humidity (RH) conditions. *P*_*RH*_, RH effect; *P*_*T*_, temperature effect; *P*_*ART*_, after-ripening time. *P_*RH*_*_×_ *_*T*_*, RH × temperature interaction effect; *P_*RH*_*_×_ *_*ART*_*, RH × after-ripening time interaction effect; *P_*T*_*_×_ *_*ART*_*, temperature × after-ripening time interaction effect; *P_*RH*_*_×_ *_*T*_*_×_ *_*ART*_*, RH × temperature × after-ripening time. Error bars represent standard error based on three biological replicates. Asterisks denote significant differences using Student’s *t*-test, *P < 0.05, **P < 0.01, and ***P < 0.001. ns stands for not significant.

The starch content of kernels rapidly decreased under the four treatments during the after-ripening stage (Figure 4A). The starch content of the kernels under the HH treatments was significantly lower than those under the LH treatments (Figure 4A). The soluble sugar content and the soluble protein content of the kernels dramatically increased under the four treatments during the after-ripening stage (Figures 4B, C). There was no significant difference in the oil content of kernels between the T20-HH and T30-HH treatments during ripening time, except for 16 d of after-ripening time (Figure 4D). There were significant negative correlations between starch and soluble sugar, soluble protein, and oil content (Table 1).

**TABLE 1:** Pearson correlation coefficients of starch content, soluble sugar, soluble protein, and oil content of *T. grandis* cv. ‘Merrillii’ nuts during post-harvest ripening at different temperature and relative humidity conditions.

**Parameters**	**Starch**	**Soluble sugar**	**Soluble protein**	**Oil**
Starch	1			
Soluble sugar	−0.705**	1		
Soluble protein	−0.770**	0.575**	1	
Oil	−0.797**	0.585**	0.754**	1

Note: The values are the correlation coefficients. – represents a negative correlation. Asterisks denote significant differences using Student’s *t*-test, **P < 0.01.

### Effects of Post-harvest Ripening Conditions on Fatty Acid Composition and UFA/SFA Ratio in *T. grandis* Nuts

**FIGURE 5:**
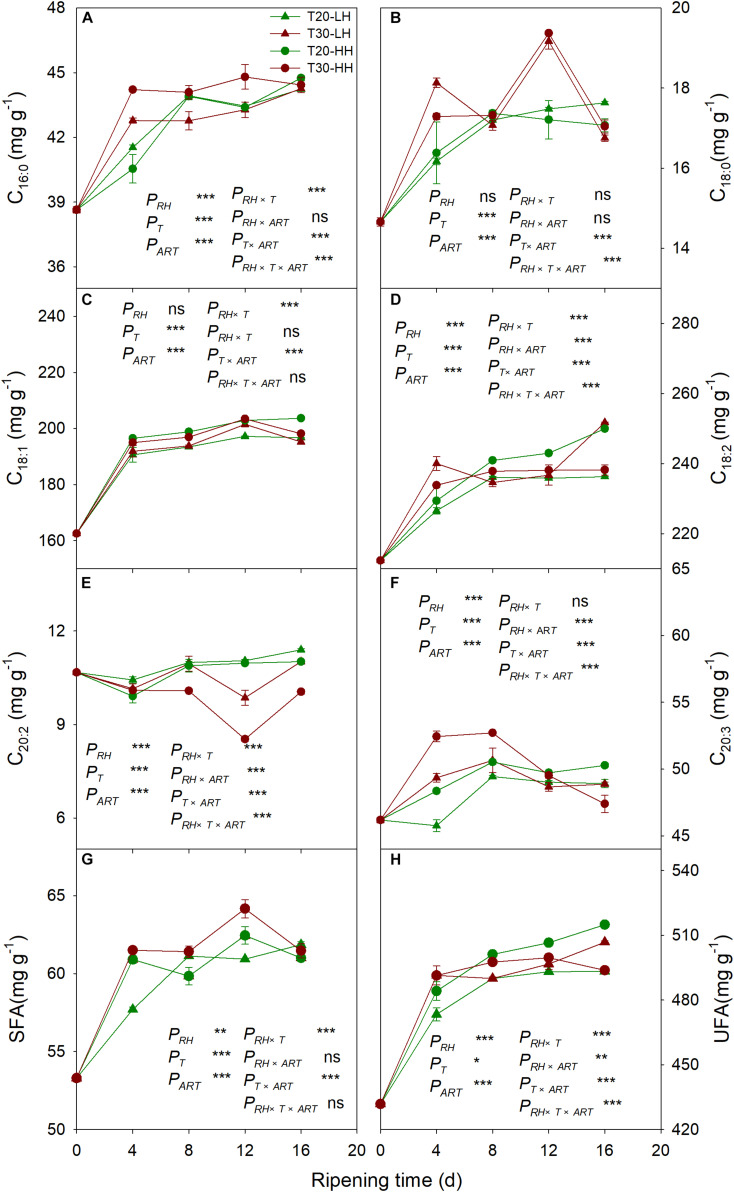
The palmitic acid (C_16:0_) content **(A)**, oleic acid (C_18:1_) content **(B)**, eicosadienoic acid (C_20:2_) content **(C)**, stearic acid (C_18:0_) content **(D)**, linoleic acid (C_18:2_) content **(E)**, sciadonic acid (C_20:3_) content **(F)**, SFA content **(G)**, and UFA content **(H)** in *T. grandis* cv. ‘Merrillii’ nuts during post-harvest ripening at different temperature and relative humidity (RH) conditions. *P*_*RH*_, RH effect; *P*_*T*_, temperature effect; *P*_*ART*_, after-ripening time. *P_*RH*_*_×_ *_*T*_*, RH × temperature interaction effect; *P_*RH*_*_×_ *_*ART*_*, RH × after-ripening time interaction effect; *P_*T*_*_×_ *_*ART*_*, temperature × after-ripening time interaction effect; *P_*RH*_*_×_ *_*T*_*_×_ *_*ART*_*, RH × temperature × after-ripening time. Error bars represent standard error based on three biological replicates. Asterisks denote significant differences using Student’s *t*-test, *P < 0.05, **P < 0.01, and ***P < 0.001. ns stands for not significant.

**FIGURE 6:**
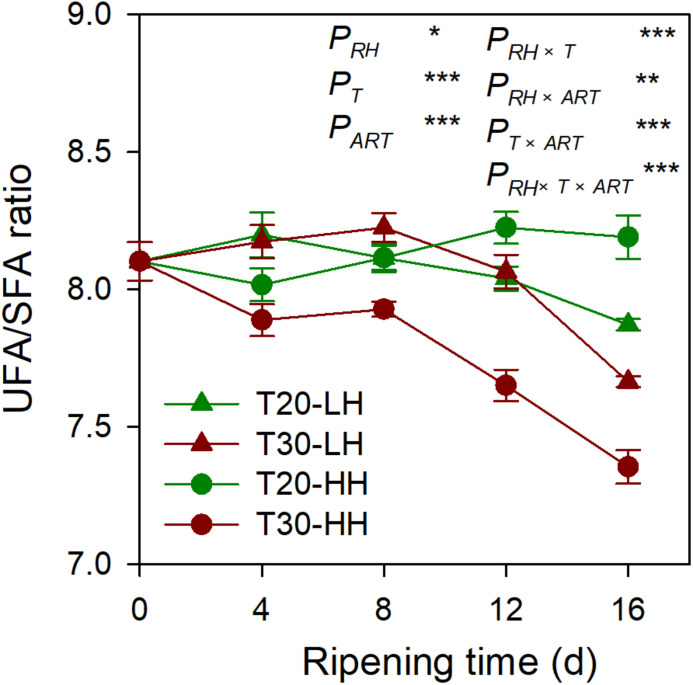
The UFA/SFA ratio of *T. grandis* cv. ‘Merrillii’ nuts during post-harvest ripening at different temperature (RT) and relative humidity (RH) conditions. Note: T20-LH, at 20°C and 70% RH; T30-LH, at 30°C and 70% RH; T20-HH, at 20°C and 90% RH; T30-HH, at 30°C and 90% RH. *P*, RH effect; *P*_*T*_, temperature effect; *P*_*ART*_, after-ripening time. *P_*RH*_*_×_ *_*T*_*, RH × temperature interaction effect; *P_*RH*_*_×_ *_*ART*_*, RH × after-ripening time interaction effect; *P_*T*_*_×_ *_*ART*_*, temperature × after-ripening time interaction effect; *P_*RH*_*_×_ *_*T*_*_×_ *_*ART*_*, RH × temperature × after-ripening time. Error bars represent standard error based on three biological replicates. Asterisks denote significant differences using Student’s *t*-test, *P < 0.05, **P < 0.01, and ***P < 0.001. ns stands for not significant.

Six primary fatty acids were identified in *T. grandis* kernel oil during after-ripening storage (Figures 5A–F). The main fatty acids were oleic acid (C_18:1_), linoleic acid (C_18:2_), and sciadonic acid (C_20:3_), which accounted for more than 87% of total fatty acid. Meantime, palmitic acid (C_16:0_), stearic acid (C_18:0_), and eicosadienoic acid (C_20:2_) were also determined to be present in small amounts. The UFA content showed an increasing trend under the T20-HH treatment during the after-ripening stage. Also, the UFA content under the T30-HH treatment showed an increased trend from 4 to 12 d of after-ripening time, followed by a decreased trend from 12 to 16 d of after-ripening time (Figures 5G, H). Under the T30-HH treatment, the UFA content was significantly lower than that of the T20-HH treatment from 12 to 16 d of after-ripening time (Figures 5G, H). The UFA/SFA ratio showed a decreasing trend under the three treatments, except for the T20-HH treatment during the after-ripening process (Figure 6).

### Effects of Post-harvest Ripening Conditions on Rancidity-Associated Enzymes and Rancidity Development in *T. grandis* Nuts

**FIGURE 7:**
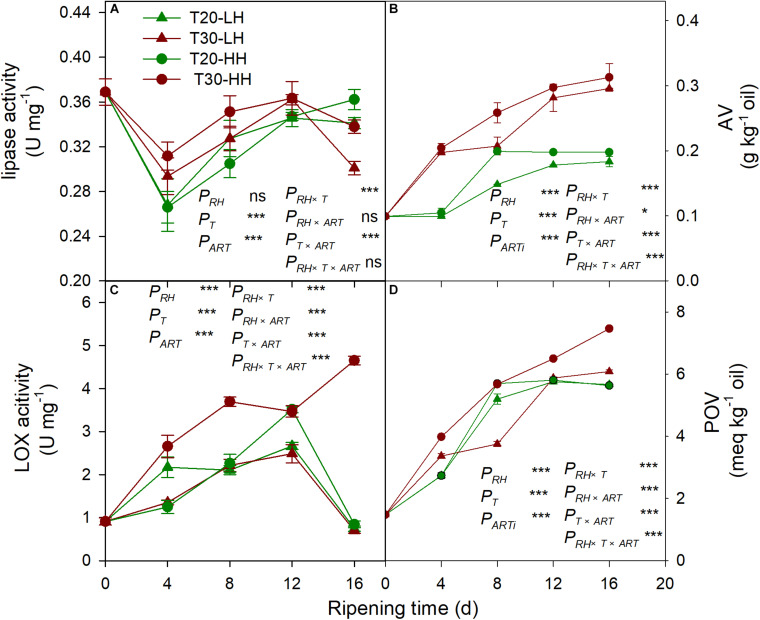
Activities of lipase activity **(A)**, AV **(B)**, LOX **(C)** and POV **(D)** in *Torreya grandis* cv. ‘Merrillii’ nuts during after-harvest ripening at different temperature and relative humidity (RH) conditions. *P*_*RH*_, RH effect; *P*_*T*_, temperature effect; *P*_*ART*_, after-ripening time. *P_*RH*_*_×_ *_*T*_*, RH × temperature interaction effect; *P_*RH*_*_×_ *_*ART*_*, RH × after-ripening time interaction effect; *P_*T*_*_×_ *_*ART*_*, temperature × after-ripening time interaction effect; *P_*RH*_*_×_ *_*T*_*_×_ *_*ART*_*, RH × temperature × after-ripening time. Error bars represent standard error based on three biological replicates. Asterisks denote significant differences using Student’s *t*-test, *P < 0.05, **P < 0.01, and ***P < 0.001. ns stands for not significant.

The lipase activity under the T20-HH treatment significantly increased from 4 to 12 d, then maintained stable until 16 d of after-ripening time, while the lipase activity under the T30-HH treatment showed an increasing trend from 4 to 12 d, followed by a decreasing trend from 12 to 16 d of after-ripening time (Figure 7A). The nuts under the T30-HH treatment showed significantly higher AV compared with the samples under the T20-HH treatment from 4 to 16 d of after-ripening time (Figure 7B). The LOX activity under the T20-HH treatment showed an increasing trend from 4 to 12 d, followed by a decreasing trend from 12 to 16 d of after-ripening time (Figure 7C). The LOX activity under the T30-HH treatment exhibited a significant increasing trend during storage stages. The LOX activity under the T30-HH treatment was significantly higher than that under the T20-HH treatment from 4 to 16 d of after-ripening time, except for no significant differences between them on day 12 (Figure 7C). The nuts under the T30-HH treatment showed a significantly higher POV compared with the samples under the T20-HH treatment from 12 to 16 d of after-ripening time (Figure 7D). The LOX activity displayed distinct positive correlations with AV and POV (Table 2).

**TABLE 2:** Pearson correlation coefficients of AV, POV, lipase activity, LOX activity, the secondary oxidation products (MDA, O${}_{2^{\cdot }}-$ and H_2_O_2_ content), and the antioxidant enzymes (SOD, POD, and CAT activity) of *T. grandis* cv. ‘Merrillii’ nuts during post-harvest ripening at different temperature and relative humidity conditions.

**Parameters**	**AV**	**POV**	**lipase activity**	**LOX activity**	**MDA**	**O_2_^–^**	**H_2_O_2_**	**SOD activity**	**POD activity**	**CAT activity**
AV	1									
POV	0.746**	1								
lipase activity	0.222	0.309*	1							
LOX activity	0.443**	0.484**	0.144	1						
MDA	0.853**	0.727**	0.339*	0.330*	1					
O_2_^–^	0.538**	0.729**	0.256	0.638**	0.549**	1				
H_2_O_2_	0.627**	0.583**	0.1	−0.009	0.500**	0.329*	1			
SOD activity	−0.548**	−0.641**	−0.263	0.114	−0.571**	−0.259	−0.576**	1		
POD activity	−0.397**	−0.171	−0.227	−0.092	−0.365**	−0.137	−0.366**	0.495**	1	
CAT activity	−0.14	−0.138	−0.366**	0.449**	−0.194	0.248	−0.398**	0.554**	0.123	1

Note: The values are the correlation coefficients. ns, correlation is not significant at the 0.05 level. Asterisks denote significant differences using Student’s *t*-test, *P < 0.05 and **P < 0.01.

### Effects of Post-harvest Ripening Conditions on the Relative Expression of *Lipase* and *LOX2* and Its Relationship With Lipase and LOX Activity in *T. grandis* Nuts

**FIGURE 8:**
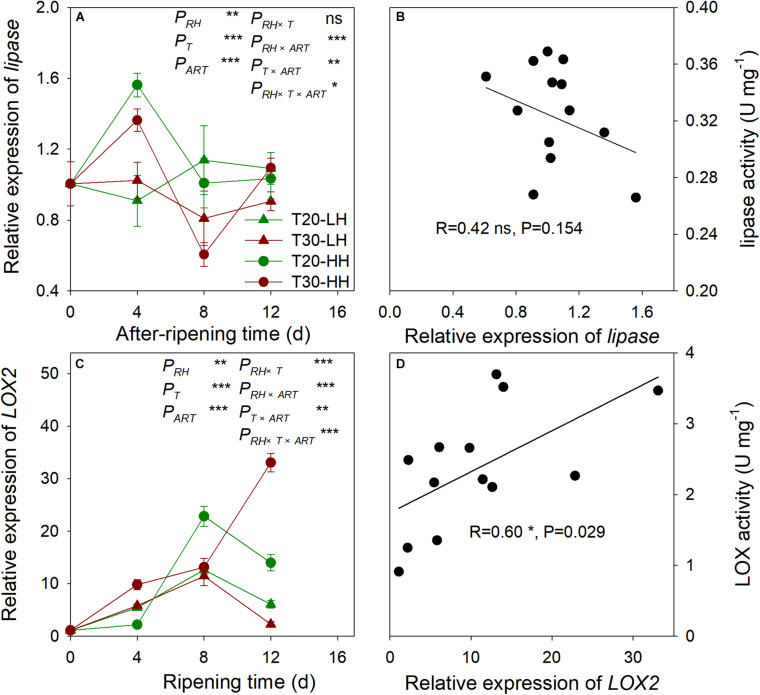
The relative expression of *lipase* **(A)**, *LOX2* **(C)**, and the correlations between the relative expression of *lipase* and lipase activity **(B)**, *LOX2* and LOX activity **(D)** of *T. grandis* cv. ‘Merrillii’ nuts during post-harvest ripening at different temperature and relative humidity (RH) conditions. *P*_*RH*_, RH effect; *P*_*T*_, temperature effect; *P*_*ART*_, after-ripening time. *P_*RH*_*_×_ *_*T*_*, RH × temperature interaction effect; *P_*RH*_*_×_ *_*ART*_*, RH × after-ripening time interaction effect; *P_*T*_*_×_ *_*ART*_*, temperature × after-ripening time interaction effect; *P_*RH*_*_×_ *_*T*_*_×_ *_*ART*_*, RH × temperature × after-ripening time. Error bars represent standard error based on three biological replicates. Asterisks denote significant differences using Student’s *t*-test, *P < 0.05, **P < 0.01, and ***P < 0.001. ns stands for not significant.

The relative expression of *lipase* remained constant under the T20-HH treatment between 8–16 d of ripening time, whereas it exhibited a significant increase under the T30-HH treatment. No significant correlation was found between lipase activity and the relative expression of *lipase* (Figure 8). The relative expression of *LOX2* showed an increasing trend under the T30-HH treatment from 4 to 12 d of after-ripening time, whereas the relative expression of *LOX2* significantly increased under the T20-HH treatment from 4 to 8 d of after-ripening time, then considerably decreased at day 12 of the after-ripening time (Figure 8). The relative expression of *LOX2* was linearly and positively correlated with LOX activity (Figure 8).

### Effects of Post-harvest Ripening Conditions on Lipid Peroxidation Products in *T. grandis* Nuts

**FIGURE 9:**
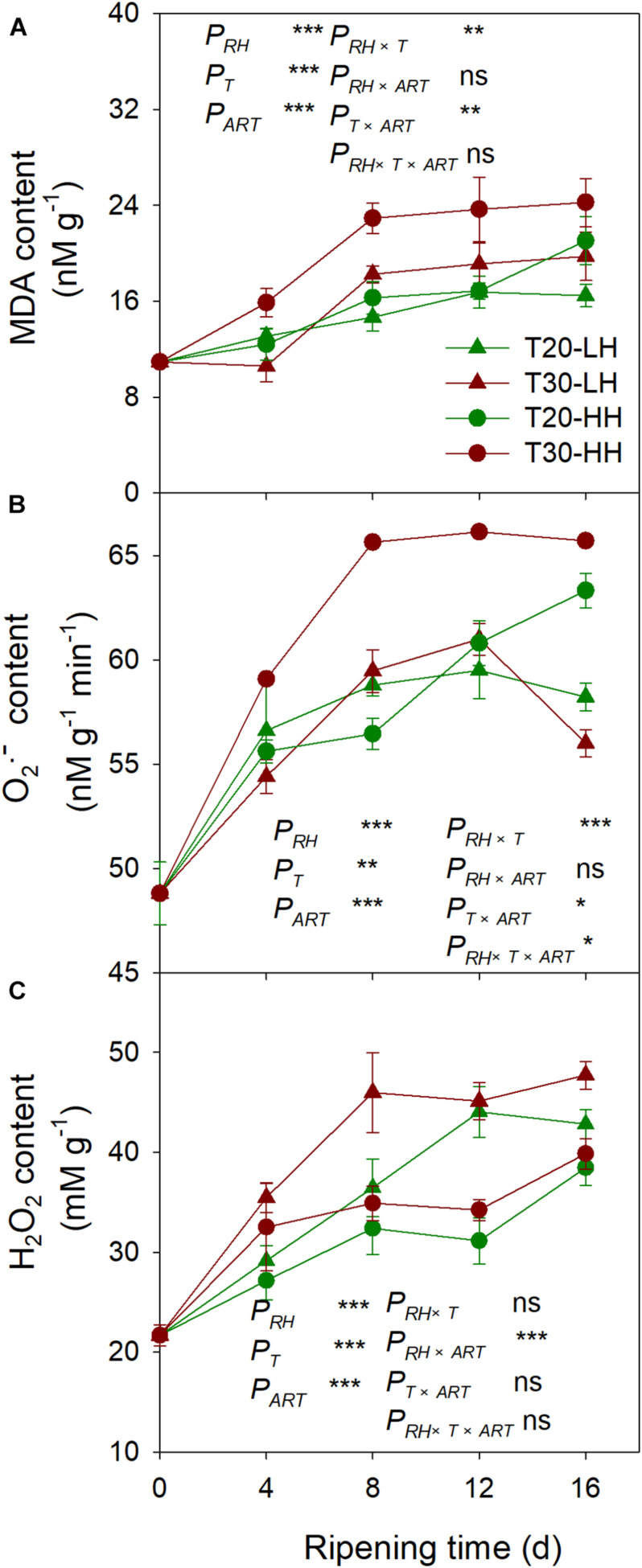
MDA content **(A)**, production rate of O_2_^–^ **(B)**, and H_2_O_2_ content **(C)** in *T. grandis* cv. ‘Merrillii’ nuts during post-harvest ripening at different temperature (RT) and relative humidity (RH) conditions. *P*_*RH*_, RH effect; *P*_*T*_, temperature effect; *P*_*ART*_, after-ripening time. *P_*RH*_*_×_ *_*T*_*, RH × temperature interaction effect; *P_*RH*_*_×_ *_*ART*_*, RH × after-ripening time interaction effect; *P_*T*_*_×_ *_*ART*_*, temperature × after-ripening time interaction effect; *P_*RH*_*_×_ *_*T*_*_×_ *_*ART*_*, RH × temperature × after-ripening time. Error bars represent standard error based on three biological replicates. Asterisks denote significant differences using Student’s *t*-test, ^∗^*P* < 0.05, ^∗∗^*P* < 0.01, and ^∗∗∗^*P* < 0.001. ns stands for not significant.

The MDA content under the T30-HH treatment was significantly higher than that of the T20-HH treatment from 4 to 12 d of after-ripening time, and no significant differences were observed on day 16. However, there were no significant differences in either the O_2_− or the H_2_O_2_ content between the T20-HH and T30-HH treatment from 12 to 16 d of after-ripening time (Figure 9). The LOX activity displayed distinct positive correlations with the content of MDA and O_2_− (Table 2).

### Effects of Post-harvest Ripening Conditions on Antioxidant Enzymes Activities in *T. grandis* Nuts

**FIGURE 10:**
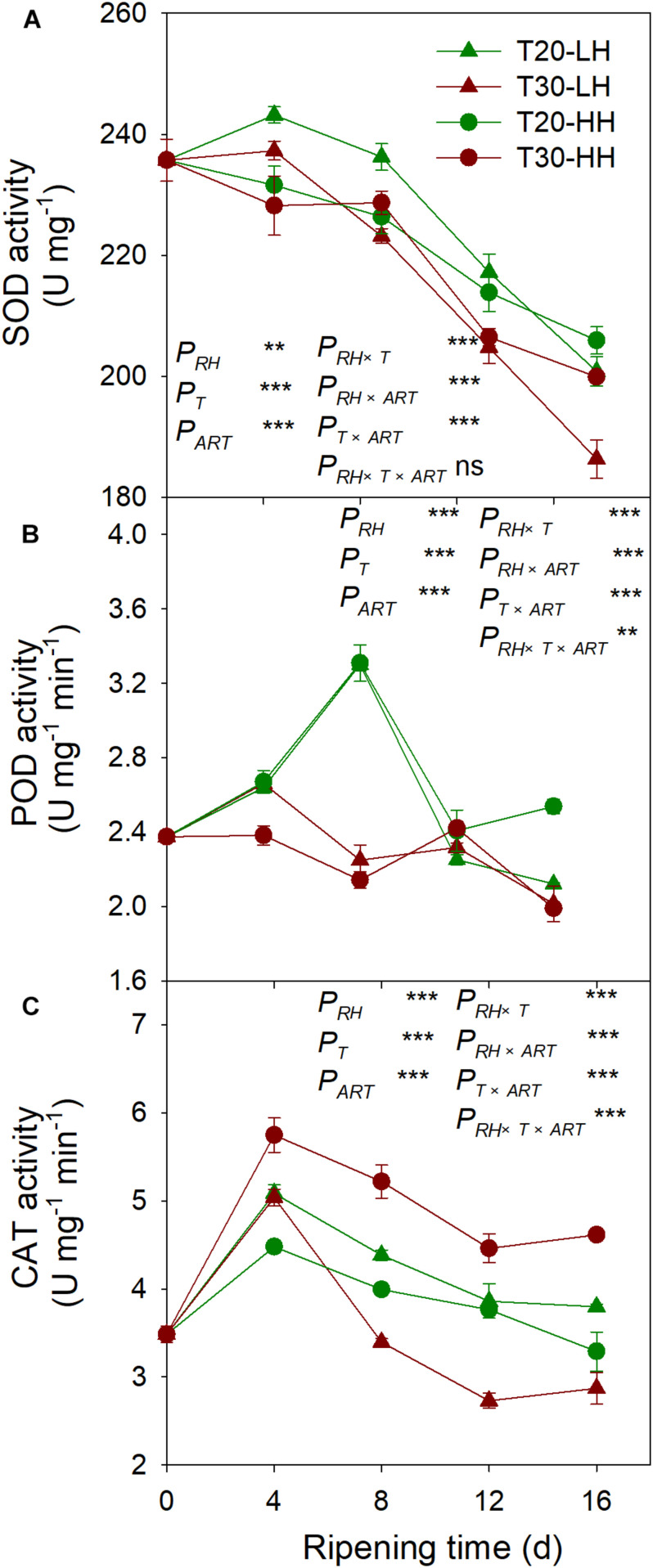
Activities of SOD **(A)**, POD **(B)**, and CAT **(C)** of *T. grandis* cv. ‘Merrillii’ nuts during post-harvest ripening at different temperature (RT) and relative humidity (RH) conditions. *P*_*RH*_, RH effect; *P*_*T*_, temperature effect; *P*_*ART*_, after-ripening time. *P_*RH*_*_×_ *_*T*_*, RH × temperature interaction effect; *P_*RH*_*_×_ *P*_*ART*_, RH × after-ripening time interaction effect; *P_*T*_*_×_ *_*ART*_*, temperature × after-ripening time interaction effect; *P_*RH*_*_×_ *_*T*_*_×_ *_*ART*_*, RH × temperature × after-ripening time. Error bars represent standard error based on three biological replicates. Asterisks denote significant differences using Student’s *t*-test, ^∗^*P* < 0.05, ^∗∗^*P* < 0.01, and ^∗∗∗^*P* < 0.001. ns stands for not significant.

The SOD activity under the four treatments showed a declining trend from 8 to 16 d of after-ripening time (Figure 10A). The POD activity under the T20-HH treatment significantly increased from 12 to 16 d of ripening time, whereas it significantly decreased under the T30-HH treatment (Figure 10B). CAT activity was significantly higher under the T30-HH treatment than the T20-HH treatment during the ripening process (Figure 10C). The activities of SOD and POD were negatively correlated with MDA and H_2_O_2_ content, respectively (Table 2).

## Discussion

### Promotion of the After-Ripening Process of *T. grandis* Nuts Under HH Treatments

In practice, the time required for after-ripening of *T. grandis* nuts is judged by the color change of the interior seed coat; the ripening is considered to be complete when the interior seed coat in *T. grandis* nuts turns completely dark (Li et al., 2005; Shan et al., 2019). The internal seed coat of *T. grandis* nuts is rich in phenolic compounds (such as tannins), which are highly astringent and bitter compounds (Mei et al., 2017). Numerous studies have shown that color changes of seed coats are the result of polyphenol oxidase enzymes, which oxidize their phenolic substrates to brown compounds (Karadeniz et al., 2000; Chazarra et al., 2001). For example, in *Acer buergerianum*, higher humidity treatment accelerated polyphenol oxidation of wood during the drying process (Gao et al., 2004). Therefore, the higher RH treatments (T20-HH and T30-HH) might accelerate the oxidation reaction of polyphenol compounds in *T. grandis* nuts.

The moisture content in the kernel was decreased during the after-ripening stage. Usually, the kernel dehydration is regulated by two contradictory phenomena (metabolic and physical basis) occurring simultaneously. On one hand, higher humidity enhanced dehydration because of the advanced metabolic ripening process. On the other hand, the low humidity induced faster physical dehydration. In the present data, the final outcome of these processes suggests that probably the kernel dehydration rate due to lower storage RH is more intense than the dehydration associated with ripening.

The seed coat of hazelnut became hard and fragile during dormancy after harvest, because the inclusions of seed coat were constantly consumed. Consequently, a large number of parenchyma cells disintegrated and fell off, and the remaining cells were loosely arranged and easily separated from the seed kernel (Zhao, 2017). Indeed, we found that the cell wall of interior seed degraded during the after-ripening stage. For *T. grandis* nuts, the distance between the kernel and the interior seed coat increased during the after-ripening time (Ye et al., 2017). In the present study, the distance between the kernel and the interior seed coat under HH treatments was significantly larger than that of the LH treatments, indicating that the larger increase in distance between the seed kernel and coat under HH treatments is related to its faster metabolic maturity rather than physical dehydration.

Starch is the main storage form of carbohydrates in seeds, which is hydrolyzed into sugar by amylase during the ripening process (Marriott et al., 1981). It provides raw materials and energy for protein and oil synthesis. The flavor and degree of crispness of *T. grandis* ‘Merrillii’ nut was negatively correlated with starch content and positively correlated with protein and oil content (Li et al., 2005). It has been reported that the degradation of starch in tobacco leaf at high humidity treatment was more rapid than that at low humidity, which was accompanied by higher total soluble sugar content (Qiu et al., 2004). The present study showed that the reduction of starch content was much higher under HH treatments than LH treatments (Figure 4). The starch content showed significant negative correlation with soluble sugar content, soluble protein content, and oil content, indicating better conversion of starch to soluble protein and oil content under HH treatments (Table 1). Therefore, we suggest that high RH conditions could accelerate the after-ripening process and provide better raw materials for the roasting process.

### Better Oil Quality of *T. grandis* Nuts Under the T20-HH Treatment During the Late After-Ripening Stage

The total oil content and UFA content under the T20-HH treatment were significantly higher than those under the T30-HH treatment at 16 d of after-ripening time, accompanied by higher levels of C_18:1_ and C_20:3_ (Figures 4, 5). UFA/SFA ratio is an indicator for assessing the nutritional value of walnut (Christopoulos and Tsantili, 2015). Here, the UFA/SFA ratio of *T. grandis* under the T20-HH treatment was constant from 4 to 16 d of after-ripening time and was significantly higher than that under the T30-HH treatment, indicating that the kernels under the T20-HH treatment maintained high and stable nutritional value during the after-ripening stage.

However, the extracted oil of the nut is very susceptible to deterioration due to UFA oxidation (Braddock et al., 1995). In nuts, the rancidity is the result of lipid oxidation and hydrolytic degradations (Ahmed and Young, 1982) and is used as a measure for evaluating nut quality, such as in walnuts, peanuts, almonds, and cashews (Evranuz, 1993; Buransompob et al., 2003; Onilude et al., 2010; Fu et al., 2016). The fast lipid oxidation of chestnut stored at 50–55% RH and room temperature was due to a significant reduction of UFA content (Wen et al., 2017). Accordingly, we suggest that the UFA content decreased significantly under the T30-HH treatment due to UFA oxidation.

### UFA Maintenance of *T. grandis* Oil Under the T20-HH Treatment Is Due to the Alleviation of Oxidation Rancidity During the Late After-Ripening Stage

The degradation of UFA is caused by hydrolytic and oxidative rancidity (Buransompob et al., 2003). Lipase is the first enzyme to act on lipid groups and hydrolyzes ester bonds (Chen, 2008). LOX catalyzed the oxidation of UFA to peroxides and hydroperoxides that were oxidized to oxy-radicals and aldehyde (Rogiers et al., 1998). AV is often used as an indicator of hydrolytic rancidity by lipase (Kirk and Sawyer, 1991). Peroxides are intermediates of the auto-oxidation process of UFA, and POV is often selected as an indicator for assessing the degree of primary oxidative rancidity of lipid (Caponio et al., 2005; Fu et al., 2016). Cashew kernel oil increased in lipid rancidity according to its POV and AV as the storage RH increased from 70% to 90% (Onilude et al., 2010). Compared with higher temperature treatments (15°C and 25°C), the rancidification process of raw *Trichosanthes kirilowii* oil was delayed under lower temperature treatments (0°C and 5°C) as indicated by significant lower POV and AV (Zhou et al., 2010). In the present study, there is a decrease in lipase activity and accompanied by a slight increase in the AV of *T. grandis* oil under the T30-HH treatment from 12 to 16 d of after-ripening time, indicating that the UFA degradation of kernel oil was not caused by hydrolytic rancidity at the late after-ripening stage. It has been reported that rancidity in walnut and almond kernels tends to be oxidative rancidity rather than hydrolytic rancidity, as indicated by fatty acid values and peroxide values (Buransompob et al., 2003). Numerous studies have shown that the POV increases gradually in perilla oil and walnut oil with increasing storage temperature (Wang et al., 2010; Vaidya and Eun, 2013; Christopoulos and Tsantili, 2015). Our results showed that the POV and LOX activity under the T30-HH treatment increased significantly by 14.9% and 32.7% from 12 to 16 d of after-ripening time, and this was accompanied by a significant positive correlation between the LOX activity and POV, indicating that primary oxidation rancidity induced by LOX activity might be the main reason for UFA degradation under the T30-HH treatment during the late after-ripening stage.

Furthermore, we observed a positive correlation between LOX activity and the relative expression of the *LOX2* gene; however, no significant correlation was found between lipase activity and the relative expression of *lipase*, indicating that more oxidative rancidity under the T30-HH treatment was significantly induced by up-regulation of *LOX2* gene at the late after-ripening stage. Previous studies revealed that the transgenic rice seed with down-regulation of *LOX2* gene using RNAi technology reduced seed quality deterioration during storage (Gayen et al., 2014). This was consistent with our result that *LOX2* gene expression and LOX activity were significantly reduced under the T20-HH treatment from 12 to 16 d of ripening time. Above all, we suggest that down-regulation of *LOX2* gene expression and LOX activity of *T. grandis* oil under the T20-HH treatment could alleviate the primary oxidation rancidity of lipids and was associated with better maintenance of oil quality.

Malondialdehyde, as a secondary final product of UFA oxidation, is a useful marker of edible lipid peroxidation (Rogiers et al., 1998). It has also been reported that lipase-derived fatty acids are utilized by *LOX* to generate O_2_− and H_2_O_2_ (Lynch and Thompson, 1984). In our present study, we also found that LOX activity was significantly positively correlated with MDA and O_2_− content of kernels (Table 2). The higher activities of SOD, CAT, AsA-POD (Ascorbic acid peroxidase), and G-POD (Guaiacol peroxidase) in ultra-dried seeds could alleviate the lipid peroxidation and maintain the membrane system (Ren, 2001). In the present study, significant negative correlations were detected between antioxidant enzymes (SOD and POD) and secondary oxidation products (MDA and H_2_O_2_) (Table 2), indicating that the kernel’s SOD and POD would effectively scavenge the secondary oxidation products during the after-ripening stage.

## Conclusion

In the present study, we found that when *T. grandis* kernels were treated under high RH treatments (T20-HH and T30-HH), the seed coat darkened quickly, and the starch content decreased rapidly, which resulted in advancement of the after-ripening process. The UFA content significantly decreased under the T30-HH treatment, but significantly increased under the T20-HH treatment from 12 to 16 d of after-ripening time. POV, which is a measure for primary oxidation product by LOX, of *T. grandis* oil under the T30-HH treatment was significantly higher than that under the T20-HH treatment during 12–16 d of ripening time. Meanwhile, a significant increase in POV and LOX activity and *LOX2* expression was detected under the T30-HH treatment from 12 to 16 d of ripening time. Our results also showed a significant positive correlation between LOX and POV activity and secondary oxidation products (MDA and O_2_−). Therefore, the oxidative rancidity is the primary type of lipid oxidation under the T30-HH treatment, which caused oil quality deterioration. Finally, of the four treatments tested, we suggest that the T20-HH treatment is the most optimal set of conditions; therefore, setting the temperature at 20°C and the RH at 90% can be a promising way to improve the oil quality of *T. grandis* nuts during post-harvest ripening.

## Data Availability Statement

All datasets presented in this study are included in the article/Supplementary Material.

## Author Contributions

LS, YH, and JW designed the work. ZZ and HJ ran the experiments and performed the data analysis and statistics. ZZ, HJ, JS, WY, MZ, WD, LS, YH, and JW wrote and reviewed the manuscript. All authors contributed to the article and approved the submitted version.

## Conflict of Interest

The authors declare that the research was conducted in the absence of any commercial or financial relationships that could be construed as a potential conflict of interest.
