# Supplementary material for: Effect of Temperature and Humidity on Oil Quality of Harvested Torreya grandis cv. Merrillii Nuts During the After-Ripening Stage
Source: Front Plant Sci. 2020 Oct 23;11:573681. doi: 10.3389/fpls.2020.573681 (PMC7644874; doi:10.3389/fpls.2020.573681)
Supplement: Supplementary file 1 [file Table_1.DOCX]

Table S1 Primers used in PCR analysis of rarget gene

| Gene | Gene ID | Primers (5’-3’) |
| --- | --- | --- |
| *ACTIN* |  | F:CGGCACACTTGAAGGGAGGT |
|  |  | R:GCCAAAGGAGCGAGGCAATTAG |
| *lipase* | *Tg*lipase_1066989 | F:CAGCGGACGTATCACCATTTTC |
|  |  | R:TCACCAGAACAGTCGCAAATCT |
| *LOX2* | *Tg*LOX2_1025506 | F:AAGCTTGATTAATGGAGGCGGA |
|  |  | R:TTTAGTGGGATCCTCAACTGCC |
